# Association of maternal epilepsy with perinatal outcomes, and an exploration of prenatal antiseizure medication: A population‐based retrospective cohort study

**DOI:** 10.1111/epi.18484

**Published:** 2025-06-16

**Authors:** Paolo Pieirino Mazzone, Jacqueline Stephen, Christopher J. Weir, Sohinee Bhattacharya, Mairead Black, Richard F. M. Chin

**Affiliations:** ^1^ Muir Maxwell Epilepsy Centre University of Edinburgh Edinburgh UK; ^2^ Child Life and Health, Centre for Inflammation Research University of Edinburgh Edinburgh UK; ^3^ Edinburgh Clinical Trials Unit, Usher Institute University of Edinburgh Edinburgh UK; ^4^ Aberdeen Centre for Women's Health Research, The Institute of Applied Health Sciences University of Aberdeen Aberdeen UK; ^5^ Royal Hospital for Children and Young People Edinburgh UK

**Keywords:** epilepsy, maternal, neonatal, perinatal, pregnancy

## Abstract

**Objective(s):**

Pregnancy in women with epilepsy is associated with adverse perinatal outcomes, but debate remains as to role of epilepsy or of prenatal antiseizure medication (ASM), or both. We aimed to investigate the association between maternal epilepsy and perinatal outcomes, and to explore the role of prenatal ASM exposure.

**Methods:**

Retrospective population‐based cohort study using linked routinely‐collected health data (Scotland, 2009–2021). We included women with at least one singleton pregnancy. Our exposures included (1) maternal epilepsy, identified using International Classification of Diseases, Tenth Revision codes; (2) prenatal ASM, defined as dispensed within the prenatal period. We conducted multi‐level multiple regressions accounting for covariates and multiple pregnancies comparing: (1) women with (WWE) and without epilepsy (WWoE), (2) all pregnancies exposed and unexposed to prenatal ASMs, and (3) individual prenatal ASM monotherapy vs without ASM.

**Results:**

A total of 629 200 pregnancies occurred from 2009–2021 (WWE = 2022, WWoE = 627 178; with ASM = 4406, without ASM = 624 794). WWE had increased odds of preterm birth, induced labor, cesarean section, preeclampsia, neonatal intensive care unit admission, low birth weight, and 5‐min Apgar score <7. In fully adjusted models, only induced labor adjusted odds ratio aOR [95 CI]b 1.17 (95% confidence interval [CI]: 1.02–1.34) remained associated. Compared to women without ASM, those with ASM had increased odds of preterm birth (aOR 1.47, 95% CI: 1.25–1.74), induced labor (1.38, 1.25–1.52), cesarean section (1.14, 1.01–1.27), neonatal congenital conditions (1.34, 1.04–1.73), neonatal intensive care unit admission (1.54, 1.33–1.78), and low birthweight (1.47, 1.23–1.75). Increased odds were also observed for specific ASM monotherapies.

**Significance:**

Maternal epilepsy is associated with many adverse perinatal outcomes, but most are driven by prenatal ASM exposure. We postulate that joint comprehensive care between obstetricians and epileptologists or other specialists who prescribe ASMs could improve perinatal outcomes. Research into models of care and prescribers and the effect on outcomes is needed.

**Key points**

- There is uncertainty about the association of maternal epilepsy with perinatal outcomes, and the role of prenatal antiseizure medications (ASMs).
- In this study, maternal epilepsy was associated with several adverse perinatal outcomes, but most were driven by prenatal ASM exposure.
- Managing epilepsy and/or ASMs in pregnancy requires personalized expert‐led care and counseling.

## 1 INTRODUCTION

In the UK, an estimated 131 000 women with epilepsy (WWE) are of childbearing age.^1^ Adequate pregnancy planning and management of WWE, particularly around antiseizure medication (ASM) choice and timing, balanced against perinatal risks is needed to optimize outcomes for mother and child.

A recent systematic review and meta‐analysis comparing perinatal outcomes for WWE and women without epilepsy (WWoE) showed that WWE are at increased odds of adverse perinatal outcomes such as, but not restricted to, maternal death and small for gestational age offspring,^2^ and that the odds of adverse perinatal outcomes were higher with greater use of ASM. Although the systematic review was able to provide up to date and more precise estimates for adverse outcomes, it was unable to account for some potential confounders, such as maternal age,^3^, ^4^ socioeconomic deprivation,^5^, ^6^, ^7^ and pre‐existing conditions.^8^, ^9^ Furthermore, the systematic review^2^ was unable to examine prenatal ASM exposure WWE compared to WWoE. This is relevant because ASM can be used for reasons other than seizure control and in conditions other than epilepsy such as migraine, psychiatric disorders, and neuropathic pain.^10^ Thus, WWoE included in the systematic review could have been exposed to prenatal ASM even though they did not have seizures/epilepsy.

Studies investigating ASM use in WWE during pregnancy tend to be on specific individual ASMs and often do not adjust for potential clinical and sociodemographic confounders.^11^, ^12^, ^13^, ^14^, ^15^, ^16^, ^17^, ^18^, ^19^, ^20^, ^21^, ^22^, ^23^, ^24^ A recent Cochrane review on monotherapy ASM treatment of epilepsy in pregnancy and congenital malformation outcomes in the child found an increased risk of neonatal congenital conditions with specific ASMs.^13^ It was not within the scope of the Cochrane review to examine ASM exposure in pregnancy in WWoE. After the Cochrane review was published, a large, Nordic, population‐based cohort study investigating ASM exposure in pregnant women (irrespective of whether they had epilepsy or not) and fetal growth, found an increased risk of small for gestational age neonates in both the overall population and in WWE who were exposed to prenatal carbamazepine, oxcarbazepine, clonazepam, and topiramate monotherapies.^17^ However, the effects on other perinatal outcomes remain unknown. Although the authors were able to adjust for several potential confounders, they were unable to account for others such as maternal socioeconomic deprivation, body mass index (BMI), or illicit drugs, or their substitute or drug misuse during pregnancy.^5^, ^6^, ^7^, ^9^, ^25^

Therefore, it is unclear after adjusting for potential confounders to what extent the increased risk of adverse perinatal outcomes in WWE is due to ASM exposure, epilepsy itself, or a combination. If any adverse outcomes are due solely or primarily to ASM exposure, this would have implications for any pregnant women taking ASMs, whether they have epilepsy or not. This study aims to investigate, after adjusting for a wide range of social and clinical factors, the association between maternal epilepsy and perinatal outcomes, and to explore the role of prenatal ASM exposure in both WWE and WWoE.

## 2 METHODS

### 2.1 Ethical and regulatory considerations

The Strengthening the Reporting of Observational Studies in Epidemiology (STROBE)^26^ and Reporting of Studies Conducted Using Observational Routinely‐Collected Data (RECORD) reporting guidelines^27^ were followed. The University of Edinburgh Research Ethics Committee provided ethical approval. Data were made available through the Public Benefit and Privacy Panel for Health and Social Care (HSC‐PBPP).

### 2.2 Study setting, design and population

This population‐based retrospective cohort study used linked routinely‐collected national health data from 2009 to 2021 in Scotland. The population included all adults (≥16 year) with at least one pregnancy.

### 2.3 Participants, variables and data sources

The National Health Service Scotland (NHSS) provides tax‐funded^28^ free‐at‐the‐point‐of‐use health care to most of the population.^29^ Patient data are obtained routinely as part of their interaction with the health services, whether this be with primary or secondary care, and whenever they access community prescriptions from pharmacies outside of hospitals. These data are managed by the Information Services Division (ISD)^30^ to provide services and advice that support the NHSS in progressing quality improvement in health and care, facilitating robust service planning and decision‐making. Therefore, it is mandatory for NHS care providers to submit their administrative data to the ISD. Hospital data are kept in the Scottish Morbidity Records (SMR), with different SMR numbers according to the type of data. For example, pregnancy records are held in SMR02 and hospital admission data are in SMR01. Community pharmacy medication dispensing information is held in the Prescribing Information Service (PIS). In order to capture ASM exposure as accurately as possible, only ASM prescriptions that were prescribed and dispensed (filled) were included. There are also birth and death data held in the Scottish Birth Register (SBR), and the National Records of Scotland, respectively.^29^ In addition, these national datasets are routinely extracted and made available to approved researchers in a secure analytical environment (Safe Haven) as linked and de‐identified research datasets.^29^ This process is managed by the electronic Data Research and Innovation Service (eDRIS) of the ISD.^29^ Linkage at the patient level is made possible using a unique patient identifier known as the Community Health Index (CHI) number. The CHI number is allocated to all patients who use the NHS in Scotland and is available on all routine administrative dataset records. Furthermore, each child born in Scotland is assigned a CHI number and this is linked from birth to the mother's CHI number.^29^ The Appendix S1 provides details on the linkage process (Figure S1), datasets, and their content (Table S1).

*Maternal epilepsy* was identified using International Classification of Diseases, Tenth Revision (ICD‐10) codes (G40, G41) from pregnancy records (SMR02), or hospital admission with an epilepsy diagnosis (SMR01) within 2 year prior to estimated conception (estimated gestation minus 2 week) through pregnancy.^31^ This definition was used so to ensure with greater likelihood that anyone coded with epilepsy would have had epilepsy as a concern for the current pregnancy (active epilepsy),^32^ rather than had epilepsy, previously such as in childhood, that had subsequently resolved (inactive epilepsy).

*Perinatal outcomes*, based on prior research^2^ and available data, were identified using ICD‐10 codes from the national health datasets described earlier, and specific outcome variables available within the datasets. For the list of perinatal outcomes and details on how they were defined, see Table S1, Appendix S1.

*Any Prenatal ASM exposure* was any ASM dispensed in the interval between 30 days before the estimated conception date and the end of pregnancy. This would have included any monotherapy, combination therapy, or dosage (Table S1, Appendix S1). ASMs were identified using British National Formulary codes (https://openprescribing.net/bnf/0408/) and based on previous research.^33^

*Prenatal exposure to individual ASM monotherapies* was defined as one individual ASM monotherapy and no other in the interval between 30 days before the estimated conception date and end of pregnancy. These medications were chosen based on clinical relevance and available data (Table S1, Appendix S1).

### 2.4 Covariates

Important covariates, and their potential to confound or modify associations between exposures and outcomes of interest, were identified through literature review and discussion with clinical experts. Our ability to measure important covariates was assessed based on our understanding of the variables available within the national administrative health datasets. The full list of covariates and details of how the covariates were defined are shown in Table S1, Appendix S1. Covariates were identified using both ICD‐10 codes and specific condition variables available within the datasets and included maternal age,^3^, ^4^ previous pregnancies,^18^, ^34^, ^35^ previous cesarean sections,^36^ socioeconomic deprivation^5^, ^6^, ^7^ using the Scottish Index of Multiple Deprivation,^37^ pre‐existing hypertensive disorders,^8^ pre‐existing diabetes,^9^ maternal BMI,^38^, ^39^ smoking,^40^ and illicit drugs or their substitute or drug misuse during pregnancy.^25^ Data on all covariates were information as obtained and recorded at the initial antenatal pregnancy episode (at booking). It is possible, therefore, that covariates that relate to history of a pre‐existing condition, like “pre‐existing hypertensive disorders,” could have included people with the condition, in this case hypertension, at any time in their life before pregnancy. Similarly, women who started smoking after at booking, would have been recorded and classified as not smoking in the current study. We dichotomized maternal age into two categories—younger than age 35 years and 35 years or older—because of the increased risk of perinatal problems in women 35 years of age or older.^3^, ^4^ We dichotomized “previous pregnancies” based on the distribution of the data to ensure that low event frequencies did not introduce model convergence issues due to small event frequencies.

Several potential covariates were considered but not included in the analyses due to an inability to measure them reliably, a high level of missing data, inadequate power to examine dose response, or they were characteristics of ASM exposure, which may influence the strength of association with outcomes rather than confounders. These included: (1) Severity of condition indicating ASM exposure, for example, seizure control in epilepsy; (2) maternal ethnicity; (3) earliest trimester of ASM exposure; (4) duration of ASM exposure during pregnancy; (5) ASM dose; (6) receipt of mono‐ or polytherapy; (7) genetic or postnatally acquired condition in baby; and (8) alcohol use in pregnancy (https://publichealthscotland.scot/publications/medicines‐in‐pregnancy‐scotland‐wide‐cohort‐study‐protocol/).

### 2.5 Statistical analysis

Data were analyzed using RStudio (Version 4.1). Repeated measures of pregnancies within mothers were accounted for using a multi‐level model. We did not correct for multiple comparisons, in part due to the increased risk of Type 2 errors, potentially masking true associations of exposures with outcomes. Instead, we focused on odds ratios (ORs) and 95% confidence intervals (CIs) as the primary measure of association, rather than relying on *p*‐value thresholds alone, to prioritize effect‐size estimation and clinical interpretation over rigid significance testing.^41^, ^42^, ^43^, ^44^, ^45^, ^46^ In addition, if there were fewer than 10 observations for a specific outcome variable, we did not model for that outcome to reduce the likelihood of overfitting. We did not investigate an interaction between prenatal ASM exposure and maternal epilepsy because several outcomes had low event frequencies. Consequently, we may not have had an adequate sample size to reliably detect an interaction, and including an interaction term could reduce model convergence.

#### 2.5.1 Maternal epilepsy

To investigate the association between maternal epilepsy and perinatal outcomes we used multi‐level binary logistic regressions, accounting for covariates and multiple pregnancies and prenatal ASM exposure. We carried out two levels of adjustment. There was an initial adjustment: (1) maternal age, previous pregnancies, fetal/neonatal sex, previous cesarean section, pre‐existing hypertensive disorders, pre‐existing diabetes, deprivation, BMI, and smoking and illicit drugs or their substitute or drug misuse during pregnancy; and (2) initial adjustment plus any prenatal ASM exposure. This allows an assessment of the extent to which any prenatal ASM exposure influences the results of perinatal outcomes in women with and without epilepsy. We did not adjust for gestational age, since it is a potential collider variable, as both maternal epilepsy and prenatal ASM exposure are associated with preterm birth. Therefore, adjusting for gestational age could have incorrectly associated neonatal outcomes with gestational age and masked the association with maternal epilepsy or prenatal ASMs.^47^

#### 2.5.2 Any prenatal ASM exposure

To explore the role of ASM in perinatal outcomes, we carried out multi‐level binary logistic regressions comparing all pregnant women (WWE and WWoE) who were taking any prenatal ASM to those not taking a prenatal ASM. We used the initial adjustment as above plus epilepsy diagnosis.

#### 2.5.3 Prenatal exposure to individual ASM monotherapies

Given the clinical interest in choice of prenatal ASM, and to allow comparison with published literature on individual ASM and outcomes, we carried out multi‐level binary logistic regressions on the women exposed to one of the five most frequently dispensed ASM monotherapies used as regular treatment of seizures in WWE. We compared all pregnant women (WWE and WWoE) on individual ASM monotherapies (topiramate, carbamazepine, levetiracetam, lamotrigine, or sodium valproate monotherapy) vs those without ASM.

### 2.6 Sensitivity analyses

Analyses using a narrower definition of congenital conditions (excluding chromosomal anomalies) were conducted for WWE vs WWoE. We extended the prenatal period by 6 months and compared results to the defined prenatal period in WWE vs WWoE. To investigate missing data, data were limited to one pregnancy per mother comparing fully adjusted complete‐case, best‐case, worst‐case, and pooled estimates from multiple imputation in WWE vs WWoE (Tables S4–S6, Appendix S1).

## 3 RESULTS

Data were available for 629 200 pregnancies in 423 894 women from 2009 to 2021. Of these pregnancies, 2022 were in WWE and 627 178 in WWoE. Among the 2022 pregnancies in WWE, 1539 (76%) had prenatal ASM exposure and 483 (24%) did not. There were 2867 (.5%) pregnancies in WWoE exposed to prenatal ASMs and 624 311 (99.5%) who were not (Table 1).

**TABLE 1:** Covariate data according to epilepsy diagnosis and prenatal antiseizure medication exposure.

Characteristic	WWoE (*N* = 627 178)	WWoE not taking any prenatal ASM (*N* = 624 311)	WWoE taking any prenatal ASM (*N* = 2867)	WWE (*N* = 2022)	WWE not on any prenatal ASM (*N* = 483)	WWE on any prenatal ASM (*N* = 1539)
Maternal age						
<35 years	492 945 (78.6)	490 668 (78.6)	2277 (79.4)	1656 (81.9)	384 (79.5)	1272 (82.7)
≥35 years	134 233 (21.4)	133 643 (21.4)	590 (20.6)	366 (18.1)	99 (20.5)	267 (17.3)
Missing	0 (.0)	0 (.0)	0 (.0)	0 (.0)	0 (.0)	0 (.0)
Previous pregnancies						
No	272 834 (43.8)	271 654 (43.8)	1180 (41.6)	842 (41.9)	221 (45.9)	621 (40.6)
Yes ≥1	350 133 (56.2)	348 476 (56.2)	1657 (58.4)	1168 (58.1)	261 (54.1)	907 (59.4)
Missing	4211 (.7)	4181 (.7)	30 (1.1)	12 (.6)	1 (.2)	11 (.7)
Previous cesarean section						
No	542 826 (86.9)	540 364 (86.9)	2462 (86.3)	1727 (85.5)	422 (87.4)	1305 (85.0)
Yes	82 046 (13.1)	81 656 (13.1)	390 (13.7)	292 (14.5)	61 (12.6)	231 (15.0)
Missing	2306 (.4)	2291 (.4)	15 (.5)	3 (.2)	0 (0)	3 (.2)
Fetal/neonatal sex						
Male	312 256 (51.4)	310 858 (51.4)	1398 (51.4)	1051 (52.7)	250 (52.9)	801 (52.7)
Female	295 160 (48.6)	293 838 (48.6)	1322 (48.6)	943 (47.3)	223 (47.1)	720 (47.3)
Missing	19 726 (3.2)	19 615 (3.1)	147 (5.1)	28 (1.4)	10 (2.1)	18 (1.2)
Deprivation^a^						
1	81 463 (13.0)	80 940 (13.0)	523 (18.3)	391 (19.3)	95 (19.7)	296 (19.2)
2	73 689 (11.8)	73 257 (11.8)	432 (15.1)	358 (17.7)	80 (16.6)	278 (18.1)
3	69 777 (11.2)	69 423 (11.1)	354 (12.4)	263 (13.0)	57 (11.8)	206 (13.4)
4	63 098 (10.1)	62 807 (10.1)	291 (10.2)	212 (10.5)	57 (11.8)	155 (10.1)
5	58 765 (9.4)	58 496 (9.4)	269 (9.4)	188 (9.3)	44 (9.1)	144 (9.4)
6	55 106 (8.8)	54 820 (8.8)	286 (10.0)	140 (6.9)	26 (5.4)	114 (7.4)
7	56 780 (9.1)	56 566 (9.1)	214 (7.5)	129 (6.4)	35 (7.3)	94 (6.1)
8	59 552 (9.5)	59 335 (9.5)	217 (7.6)	123 (6.1)	29 (6.0)	94 (6.1)
9	55 970 (8.9)	55 827 (9.0)	143 (5.0)	119 (5.9)	31 (6.4)	88 (5.7)
10	51 208 (8.2)	51 073 (8.2)	135 (4.7)	98 (4.8)	28 (5.8)	70 (4.5)
Missing	1770 (.3)	1767 (.3)	3 (.1)	1 (.1)	1 (.2)	0 (0)
Pre‐existing hypertensive disorder						
No	624 916 (99.6)	622 062 (99.6)	2854 (99.5)	2007 (99.3)	478 (n.c.)	1529 (n.c.)
Yes	2262 (.4)	2249 (.40)	13 (.5)	15 (.7)	<10 (n.c.)	<10 (n.c.)
Missing	0 (.0)	0 (.0)	0 (.0)	0 (.0)	n.c.	n.c.
Pre‐existing diabetes						
No	622 397 (99.2)	619 570 (99.2)	2827 (98.6)	2000 (98.9)	478 (n.c.)	1522 (98.9)
Yes	4781 (.8)	4741 (.8)	40 (1.4)	22 (1.1)	<10 (n.c.)	17 (1.1)
Missing	0 (.0)	0 (.0)	0 (.0)	0 (.0)	n.c.	0 (.0)
BMI						
Normal	216 869 (43.9)	216 086 (43.9)	783 (36.0)	686 (41.3)	162 (40.0)	524 (41.7)
Obese	128 894 (26.1)	128 142 (26.1)	752 (34.6)	493 (29.6)	109 (26.9)	384 (30.5)
Overweight	134 568 (27.2)	133 994 (27.2)	574 (26.4)	421 (25.3)	112 (27.7)	309 (24.6)
Underweight	14 135 (2.9)	14 071 (2.9)	64 (2.9)	63 (3.8)	22 (5.4)	41 (3.3)
Missing	132 712 (21.2)	132 018 (21.2)	694 (24.2)	359 (17.8)	78 (16.2)	281 (18.3)
Illicit drugs or their substitute or drug misuse during pregnancy						
No	499 954 (98.3)	497 890 (98.3)	2064 (94.9)	1701 (95.0)	415 (95.2)	1286 (94.9)
Yes	8563 (1.7)	8452 (1.7)	111 (5.1)	90 (5.0)	21 (4.8)	69 (5.1)
Missing	118 661 (18.9)	117 969 (18.9)	692 (24.1)	231 (11.4)	47 (9.7)	184 (12.0)
Smoker during pregnancy						
No	494 726 (83.3)	492 781 (83.3)	1945 (72.4)	1406 (73.0)	322 (70.2)	1084 (73.9)
Yes	99 276 (16.7)	98 534 (16.7)	742 (27.6)	519 (27.0)	137 (29.8)	382 (26.1)
Missing	33 176 (5.3)	32 996 (5.3)	180 (6.3)	97 (4.8)	24 (5.0)	72 (4.7)^b^

*Note*: Data are no. (%) unless indicated otherwise.

Abbreviations: ASM, antiseizure medication; BMI, body mass index; n.c., not calculated; WWE−, women with epilepsy not taking prenatal ASM; WWE, women with epilepsy; WWE+, women with epilepsy taking any prenatal ASM; WWoE, women without epilepsy; WWoE−, women without epilepsy not taking prenatal ASM; WWoE+, women without epilepsy taking any prenatal ASM.

^a^ Deprivation based on the Scottish Index of Multiple Deprivation 2020 – Scotland level population‐weighted decile (1 = most deprived; 10 = least deprived).

^b^ Certain entries have been suppressed due to disclosure concerns.

### 3.1 WWE vs WWoE

WWE and WWoE were similar according to previous pregnancies, previous cesarean section, and fetal/neonatal sex. WWE had increased odds of being ≥35 years of age, having pre‐existing hypertension, obesity, smoking, and illicit drugs or their substitute or drug misuse during pregnancy, and reduced odds of being in the lowest deprivation decile (Table S3).

In our initial adjustment, compared to WWoE, WWE had increased odds of preterm birth, induced labor, cesarean section, preeclampsia, neonatal intensive care unit (NICU) admission, low birth weight, and 5‐min Apgar score <7 (Table 2). Furthermore, with the inclusion of any prenatal ASM in the model, only increased odds of induced labor adjusted OR (aOR) 1.17 (95% CI 1.04–1.31]) remained (Table 2). These results indicate that much of the increase in odds could be attributed to prenatal ASM exposure. No differences were seen between WWE and WWoE concerning congenital conditions (Table 2). Sensitivity analyses did not change our findings (Tables S4–S6), indicating that the defined prenatal period effectively measures prenatal ASM exposure and that missing data had minimal impact on the results.

**TABLE 2:** Perinatal outcomes for all pregnancies in women with and without epilepsy.

	WWoE (*N* = 627 178) ~reference~	WWE (*N* = 2022)	OR [95% CI]	aOR [95% CI]_a_	aOR [95 CI]_b_
Maternal and fetal outcomes					
Preterm birth	36 853 (5.9)	197 (9.7)	**1.68 [1.44, 1.97]**	**1.46 [1.21, 1.77]**	1.09 [.87, 1.37]
Induced labor	177 379 (28.3)	761 (37.6)	**1.51 [1.37, 1.66]**	**1.48 [1.32, 1.66]**	**1.17 [1.02, 1.34]**
Cesarean section	187 069 (29.8)	670 (33.1)	**1.28 [1.09, 1.51]**	**1.16 [1.02, 1.32]**	1.05 [.90, 1.23]
Stillbirth	2041 (.3)	<10 (n.c.)	n.c.	n.c.	n.c.
Gestational hypertension	12 044 (1.9)	26 (1.3)	.**68 [0.46, 1.00]**	.83 [.51, 1.37]	.83 [.51, 1.37]
Eclampsia	235 (<.1)	<10 (n.c.)	n.c.	n.c.	n.c.
Preeclampsia	7179 (1.1)	33 (1.6)	**1.42 [1.00, 2.02]**	**1.46 [1.21, 1.77]**	1.36 [.80, 2.31]
Gestational diabetes	20 118 (3.2)	84 (4.2)	**1.30 [1.02, 1.66]**	1.00 [.74, 1.34]	.95 [.68, 1.34]
APH	6199 (1.0)	22 (1.1)	1.10 [0.72, 1.68]	1.03 [.62, 1.69]	.95 [.53, 1.72]
PPH	203 980 (32.5)	698 (34.5)	1.10 [0.99, 1.22]	1.09 [.97, 1.23]	1.07 [.93, 1.24]
Placental abruption	1081 (0.2)	<10 (n.c.)	n.c.	n.c.	n.c.
Placenta previa	1450 (0.2)	<10 (n.c.)	n.c.	n.c.	n.c.
Maternal death	164 (<0.1)	<10 (n.c.)	n.c.	n.c.	n.c.
Neonatal outcomes					
Congenital conditions^a^	15 055 (2.4)	52 (2.6)	1.07 [0.81, 1.41]	1.24 [.91, 1.67]	.98 [.68, 1.41]
NICU	45 068 (7.2)	203 (10.0)	**1.43 [1.23, 1.66]**	**1.36 [1.14, 1.62]**	.97 [.79, 1.20]
LBW	31 087 (5.0)	186 (9.2)	**1.90 [1.62, 2.23]**	**1.49 [1.22, 1.82]**	1.12 [.88, 1.42]
Neonatal/infant death	838 (0.1)	<10 (n.c.)	n.c.	n.c.	n.c.
Apgar 5 min <7 (%)	16 428 (2.6)	79 (3.9)	**1.51 [1.20, 1.90]**	**1.52 [1.17, 1.97]**	1.32 [.96, 1.81]^b^

*Note*: Bold values are statistically significant.

Abbreviations: 95% CI, 95% confidence interval; aOR, adjusted odds ratio; aOR_a_, adjusted for maternal age, parity, previous cesarean sections, offspring sex, deprivation, pre‐existing hypertensive disorder, pre‐existing diabetes, BMI, illicit drugs or their substitute or drug misuse during pregnancy, smoking during pregnancy (initial adjustment); aOR_b_, initial adjustment plus any prenatal ASM; APH, antepartum hemorrhage; LBW, low birth weight; n.c., not calculated to prevent overfitting due to low event frequencies; NICU, neonatal intensive care unit admission; OR, odds ratio; PPH, postpartum hemorrhage; WWE, women with epilepsy; WWoE, women without epilepsy. Data are no. (%) unless indicated otherwise.

^a^ Congenital conditions include any congenital condition as identified using ICD‐10 codes Q00‐Q99 (please see Table S1, Appendix S1).

^b^ Certain entries have been suppressed due to disclosure concerns.

### 3.2 Prenatal ASM exposure vs. no prenatal ASM exposure

Of 629 200 pregnancies, 4406 were with ASM (WWoE = 2867; WWE = 1539) and 624 794 were without ASM (WWoE = 624 311; WWE = 483) (Table 3). Women in the with ASM and those in the without ASM groups were similar according to fetal/neonatal sex and pre‐existing hypertensive disorders. However, women who had prenatal ASM exposure were more likely to be older and more socioeconomically deprived and have potential social/clinical confounders (Table 3).

**TABLE 3:** Covariate data according to prenatal anti‐seizure medication exposure and OR of taking any ASM vs not taking any ASM, and of each of five ASM monotherapies vs not taking ASM.

Characteristic	No ASM (*N* = 624 794)	On ASM (*N* = 4406)	TPR (*N* = 391)	CBZ (*N* = 600)	LVT (*N* = 832)	LMT (*N* = 1469)	SVP (*N* = 315)
Epilepsy diagnosis							
No	624 311 (99.9)	2867 (65.1)	369 (94.4)	421 (70.2)	460 (55.3)	1012 (68.9)	215 (68.3)
Yes	483 (.1)	1539 (34.9) OR 693.85 [95% CI 622.44, 773.45]	22 (5.6)	179 (29.8)	372 (44.7)	457 (31.1)	100 (31.7)
Missing	0 (0.0)	0 (0.0)	0 (.0)	0 (.0)	0 (.0)	0 (.0)	0 (.0)
Maternal age							
<35 years	491 052 (78.6)	3549 (80.5)	325 (83.1)	416 (69.3)	727 (87.4)	1174 (79.9)	238 (75.6)
≥35 years	133 742 (21.4)	857 (19.5) OR 0.89 [95% CI 0.82, .96]	66 (16.9)	184 (30.7)	105 (12.6)	295 (20.1)	77 (24.4)
Missing	0 (0.0)	0 (0.0)	0 (0.0)	0 (0.0)	0 (.0)	0 (.0)	0 (.0)
Previous pregnancies							
No	271 875 (43.8)	1801 (41.3)	141 (36.4)	198 (33.2)	382 (46.5)	613 (42.2)	107 (34.1)
Yes ≥1	348 737 (56.2)	2564 (58.7) OR 1.11 [95% CI 1.04, 1.18]	246 (63.6)	398 (66.8)	440 (53.5)	838 (57.8)	207 (65.9)
Missing	4182 (.7)	41 (0.9)	4 (1.0)	4 (.7)	10 (1.2)	18 (1.2)	1 (.3)
Previous cesarean section							
No	540 786 (86.9)	3767 (85.8)	317 (81.7)	510 (85.3)	738 (88.9)	1244 (85.0)	258 (82.2)
Yes	81 717 (13.1)	621 (14.2) OR 1.09 [95% CI 1.00, 1.19]	71 (18.3)	88 (14.7)	92 (11.1)	219 (15.0)	56 (17.8)
Missing	2291 (.4)	18 (0.4)	3 (.8)	2 (.3)	2 (.2)	6 (.4)	1 (.3)
Fetal/neonatal sex							
Male	311 108 (51.4)	2199 (51.9)	186 (49.2)	282 (50.2)	426 (52.5)	744 (52.2)	155 (51.8)
Female	294 061 (48.6)	2042 (48.1) OR 0.98 [95% CI 0.92, 1.04]	192 (50.8)	280 (49.8)	385 (47.5)	681 (48.8)	144 (48.2)
Missing	19 625 (3.1)	165 (3.8)	13 (3.3)	38 (6.3)	21 (2.5)	44 (3.0)	16 (5.1)
Deprivation							
1	81 035 (13.0)	819 (18.6)	58 (n.c.)	85 (14.2)	203 (24.4)	233 (15.9)	72 (22.9)
2	73 337 (11.8)	710 (16.1)	64 (n.c.)	85 (14.2)	122 (14.7)	218 (14.8)	57 (18.1)
3	69 480 (11.2)	560 (12.7)	60 (n.c.)	72 (12.0)	104 (12.5)	208 (14.2)	31 (9.8)
4	62 864 (10.1)	446 (10.1)	46 (n.c.)	72 (12.0)	74 (8.9)	147 (10.0)	27 (8.6)
5	58 540 (9.4)	413 (9.4)	37 (n.c.)	57 (9.5)	72 (8.7)	142 (9.7)	29 (9.2)
6	54 846 (8.8)	400 (9.1)	34 (n.c.)	66 (11.0)	65 (7.8)	138 (9.4)	31 (9.8)
7	56 601 (9.1)	308 (7.0)	29 (n.c.)	49 (8.2)	67 (8.1)	106 (7.2)	20 (6.3)
8	59 364 (9.5)	311 (7.1)	42 (n.c.)	40 (6.7)	55 (6.6)	105 (7.1)	17 (5.4)
9	55 858 (9.0)	231 (5.2)	<10 (n.c.)	39 (6.5)	42 (5.0)	89 (6.1)	18 (5.7)
10	51 101 (8.2)	205 (4.7) OR 0.55 [95% CI 0.47, 0.63]	11 (n.c.)	34 (5.7)	28 (3.4)	83 (5.7)	13 (4.1)
Missing	1768 (.3)	3 (0.1)	n.c.	1 (.2)	0 (.0)	0 (.0)	0 (.0)
Pre‐existing hypertensive disorders							
No	622 540 (99.6)	4383 (99.5)	387 (n.c.)	595 (n.c.)	831 (n.c.)	1459 (n.c.)	310 (n.c.)
Yes	2254 (.4)	23 (0.5) OR 1.45 [95% CI 0.96, 2.19]	<10 (n.c.)	<10 (n.c.)	<10 (n.c.)	<10 (n.c.)	<10 (n.c.)
Missing	0 (.0)	0 (0.0)	n.c.	n.c.	n.c.	n.c.	n.c.
Pre‐existing diabetes							
No	620 048 (99.2)	4349 (98.7)	384 (n.c.)	583 (97.2)	826 (n.c.)	1449 (98.6)	310 (n.c.)
Yes	4746 (.8)	57 (1.3) OR 1.71 [95% CI 1.32, 2.23]	<10 (n.c.)	17 (2.8)	<10 (n.c.)	20 (1.4)	<10 (n.c.)
Missing	0 (.0)	0 (0.0)	n.c.	0 (.0)	n.c.	0 (.0)	n.c.
BMI							
Normal	216 248 (43.9)	1307 (38.1)	100 (n.c.)	178 (n.c.)	274 (42.9)	444 (38.5)	77 (31.7)
Obese	128 251 (26.0)	1136 (33.1) OR 1.41 [95% CI 1.31, 1.51]	134 (n.c.)	173 (n.c.)	171 (26.8)	356 (30.8)	99 (40.7)
Overweight	134 106 (27.2)	883 (25.7)	75 (n.c.)	110 (n.c.)	168 (26.3)	321 (27.8)	59 (24.3)
Underweight	14 093 (2.9)	105 (3.1)	<10 (n.c.)	<10 (n.c.)	25 (3.9)	33 (2.9)	8 (3.3)
Missing	132 096 (21.1)	975 (22.1)	n.c.	n.c.	194 (23.3)	315 (21.4)	72 (22.9)
Illicit drugs or their substitute or Drug Misuse during pregnancy							
No	498 305 (98.3)	3350 (94.9)	308 (n.c.)	476 (95.0)	616 (95.2)	1115 (94.2)	229 (95.0)
Yes	8473 (1.7)	180 (5.1) OR 3.16 [95% CI 2.72, 3.68]	<10 (n.c.)	25 (5.0)	31 (4.8)	69 (5.8)	12 (5.0)
Missing	118 016 (18.9)	876 (19.9)	n.c.	99 (16.5)	185 (22.2)	285 (19.4)	74 (23.5)
Smoker during pregnancy							
No	493 103 (83.3)	3029 (72.9)	277 (74.5)	458 (80.8)	571 (72.9)	998 (71.40)	201 (68.4)
Yes	98 671 (16.7)	1124 (27.1) OR 1.85 [95% CI 1.73, 1.99]	95 (25.5)	109 (19.2)	212 (27.1)	400 (28.6)	93
Missing	33 020 (5.3)	253 (5.7)	19 (4.9)	33 (5.5)	49 (5.9)	71 (4.8)	21 (6.7)^a^

*Note*: Data are no. (%) unless indicated otherwise.

Abbreviations: 95 CI, 95% Confidence interval; ASM, antiseizure medication; BMI, body mass index; CBZ, carbamazepine; LMT, lamotrigine; LVT, levetiracetam; n.c., not calculated; No ASM−, all pregnancies not exposed to prenatal ASM; On ASM, all pregnancies exposed to any prenatal ASM; OR, odds ratio; SVP, sodium valproate; TPR, topiramate.

^a^ Certain entries have been suppressed due to disclosure concerns.

In fully adjusted models, women with ASM had increased odds of adverse outcomes, including preterm birth, induced labor, cesarean section, congenital conditions, NICU, and low birth weight. These results indicate that even when accounting for potential confounders, pregnant women exposed to prenatal ASMs were at increased odds of adverse perinatal outcomes, compared to pregnant women unexposed (Table 4).

**TABLE 4:** Perinatal outcomes for all pregnancies (2009–2021) by any prenatal ASM and five individual ASM monotherapy exposure compared to no prenatal ASM exposure.

	No ASM (*N* = 624 794) ~reference~	Any ASM (*N* = 4406)	OR [95% CI]	aOR [95% CI]	aOR [95% CI] TPR (*N* = 391)	aOR [95 CI] CBZ (*N* = 600)	aOR [95 CI] LVT (*N* = 832)	aOR [95 CI] LMT (*N* = 1469)	aOR [95 CI] SVP (*N* = 315)
Maternal and fetal outcomes									
Preterm birth	36 613 (5.9)	437 (9.9)	**1.74 [1.56, 1.95]**	**1.47 [1.25, 1.74]**	1.20 [.74, 1.96] (*n* = 37)	**1.62 [1.14, 2.32]** (*n* = 62)	**1.87 [1.37, 2.55]** (*n* = 87)	.96 [.72, 1.26] (*n* = 124)	1.55 [.95, 2.54] (*n* = 31)
Induced labor	176 539 (28.3)	1601 (36.3)	**1.44 [1.35, 1.54]**	**1.38 [1.25, 1.52]**	**1.48 [1.12, 1.96]** (*n* = 142)	**1.48 [1.18, 1.86]** (*n* = 215)	1.10 [.90, 1.36] (*n* = 302)	1.12 [.96, 1.30] (*n* = 504)	**1.65 [1.20, 2.27]** (*n* = 119)
Cesarean section	186 309 (29.8)	1430 (32.5)	**1.21 [1.07, 1.37]**	**1.14 [1.01, 1.27]**	1.00 [.72, 1.39] (*n* = 131)	.98 [.75, 1.28] (*n* = 175)	**1.31 [1.04, 1.64]** (*n* = 258)	.99 [.84, 1.18] (*n* = 482)	.97 [.67, 1.40] (*n* = 104)
Stillbirth	2027 (0.3)	23 (0.5)	**1.61 [1.07, 2.43]**	1.59 [0.82, 3.07]	n.c. (*n* < 10)	n.c. (*n* < 10)	n.c. (*n* < 10)	n.c. (*n* < 10)	n.c. (*n* < 10)
Gestational hypertension	12 007 (1.9)	63 (1.4)	**0.74 [0.58, 0.96]**	0.80 [0.57, 1.13]	n.c. (*n* < 10)	**1.80 [1.02, 3.19]** (*n* = 16)	n.c. (*n* < 10)	1.12 [.70, 1.77] (*n* = 26)	n.c. (*n* < 10)
Eclampsia	234 (<0.1)	<10 (n.c.)	n.c.	n.c.	n.c. (*n* < 10)	n.c. (*n* < 10)	n.c. (*n* < 10)	n.c. (*n* < 10)	n.c. (*n* < 10)
Preeclampsia	7160 (1.2)	52 (1.2)	1.03 [0.78, 1.36]	0.79 [0.52, 1.21]	n.c. (*n* < 10)	n.c. (*n* < 10)	n.c. (*n* < 10)	n.c. (*n* < 10)	n.c. (*n* < 10)
Gestational diabetes	20 030 (3.2)	172 (3.9)	**1.21 [1.02, 1.44]**	1.07 [0.84, 1.35]	1.21 [.68, 2.16] (*n* = 21)	.97 [.57, 1.66] (*n* = 29)	1.33 [.81, 2.18] (*n* = 30)	1.09 [.75, 1.56] (*n* = 53)	.95 [.44, 2.07] (*n* = 12)
APH	6170 (1.0)	51 (1.2)	1.17 [0.88, 1.54]	1.10 [0.72, 1.67]	n.c. (*n* < 10)	n.c. (*n* < 10)	n.c. (*n* < 10)	n.c. (*n* < 10)	n.c. (*n* < 10)
PPH	203 226 (32.5)	1452 (33.0)	1.03 [0.96, 1.11]	1.02 [0.92, 1.13]	1.02 [.77, 1.36] (*n* = 126)	1.00 [.79, 1.27] (*n* = 197)	1.05 [.85, 1.29] (*n* = 271)	1.04 [.89, 1.21] (*n* = 500)	.**68 [.48, .96]** (*n* = 86)
Placental abruption	1077 (.2)	12 (.3)	1.58 [.89, 2.80]	1.26 [.54, 2.90]	n.c. (*n* < 10)	n.c. (*n* < 10)	n.c. (*n* < 10)	n.c. (*n* < 10)	n.c. (*n* < 10)
Placenta previa	1447 (.2)	<10 (n.c.)	n.c.	n.c.	n.c. (*n* < 10)	n.c. (*n* < 10)	n.c. (*n* < 10)	n.c. (*n* < 10)	n.c. (*n* < 10)
Maternal deaths	159 (<.1)	<10 (n.c.)	n.c.	n.c.	n.c. (*n* < 10)	n.c. (*n* < 10)	n.c. (*n* < 10)	n.c. (*n* < 10)	n.c. (*n* < 10)
Neonatal outcomes									
Congenital conditions^a^	14 967 (2.4)	140 (3.2)	**1.34 [1.13, 1.59]**	**1.34 [1.04, 1.73]**	1.24 [0.61, 2.53] (*n* = 14)	**1.90 [1.16, 3.10]** (*n* = 22)	.91 [.51, 1.63] (*n* = 19)	.76 [.48, 1.20] (*n* = 38)	**3.91 [2.36, 6.49]** (*n* = 24)
NICU	44 768 (7.2)	503 (11.4)	**1.66 [1.51, 1.83]**	**1.54 [1.33, 1.78]**	1.02 [0.65, 1.62] (*n* = 34)	**1.59 [1.15, 2.18]** (*n* = 68)	**1.45 [1.08, 1.94]** (*n* = 94)	1.22 [.97, 1.53] (*n* = 152)	**1.96 [1.29, 2.97]** (*n* = 43)
LBW	30 896 (5.0)	377 (8.6)	**1.79 [1.59, 2.00]**	**1.47 [1.23, 1.75]**	1.02 [0.58, 1.78] (*n* = 22)	**1.70 [1.17, 2.49]** (*n* = 55)	**1.68 [1.21, 2.32]** (*n* = 83)	.95 [.71, 1.27] (*n* = 104)	1.65 [.99, 2.75] (*n* = 28)
Neonatal/infant death	831 (.1)	<10 (n.c.)	n.c.	n.c.	n.c. (*n* < 10)	n.c. (*n* < 10)	n.c. (*n* < 10)	n.c. (*n* < 10)	n.c. (*n* < 10)
Apgar 5 min <7	16 343 (2.6)	164 (3.7)	**1.44 [1.23, 1.69]**	1.20 [.95, 1.53]	n.c. (*n* < 10)	1.05 [.59, 1.85] (*n* = 18)	1.42 [.91, 2.21] (*n* = 36)	1.00 [.68, 1.46] (*n* = 47)^b^	1.79 [.96, 3.35] (*n* = 19)

*Note*: aOR, Adjusted for maternal age, previous pregnancies, previous cesarean sections, fetal/neonatal sex, deprivation, pre‐existing hypertensive disorders, pre‐existing diabetes, epilepsy diagnosis, BMI, illicit drugs or their substitute or drug misuse during pregnancy, smoking during pregnancy. Data are no. (%) unless indicated otherwise. Bold values are statistically significant.

Abbreviations: 95% CI, 95% confidence interval; Any ASM, All pregnancies exposed to any prenatal ASM; aOR: adjusted odds ratio; APH, antepartum hemorrhage; CBZ, carbamazepine; LBW, low birth weight; LMT, lamotrigine; LVT, levetiracetam; n.c., not calculated to prevent overfitting due to low event frequencies; NICU, neonatal intensive care unit admission; No ASM, all pregnancies not exposed to prenatal ASM; OR, odds ratio; PPH, postpartum hemorrhage; SVP, sodium valproate; TPR, topiramate.

^a^ Congenital conditions include any congenital condition as identified using ICD‐10 codes Q00‐Q99 (please see Table S1, Appendix S1).

^b^ Certain entries have been suppressed due to disclosure concerns.

### 3.3 Individual ASM monotherapy vs no ASM exposure

Among 4406 pregnancies in which there was prenatal exposure to ASM (irrespective of whether the pregnant women had epilepsy or not), 391 were exposed to topiramate, 600 to carbamazepine, 832 to levetiracetam, 1469 to lamotrigine, and 315 to sodium valproate monotherapy (Table 3).

In fully adjusted models, pregnancies in which there was prenatal exposure to any of the individual five ASMs were associated with increased odds of adverse perinatal outcomes, and this varied according to ASM type. This included congenital conditions in valproate (3.91, 2.36– 6.49) and carbamazepine (1.90, 1.16–3.10), and increased odds of induced labor was observed in pregnancies with prenatal topiramate (1.48, 1.12–1.96), carbamazepine (1.48, 1.18–1.86), and valproate exposure (1.65, 1.20–2.27) (Table 4). For other outcomes, the event frequencies were low and CIs were wide, necessitating caution in interpreting results.

## 4 DISCUSSION

This population‐based retrospective cohort study highlights that: (1) pregnant women with epilepsy are more likely to experience several adverse perinatal outcomes, but after adjusting for prenatal ASM and other covariates, only increased odds of induced labor remained; (2) compared to pregnancies in which there was no prenatal ASM exposure, prenatal exposure to any ASM for any reason, and independent of whether the woman had epilepsy, was associated with increased odds of several adverse outcomes; and (3) prenatal valproate or carbamazepine exposure (but not topiramate or levetiracetam or lamotrigine) is associated with congenital conditions in offspring.

Notwithstanding our lack of data on the details of the epilepsy characteristics of WWE, to our knowledge, the current study is the first to examine outcomes of pregnant WWE, adjusting for a comprehensive social and clinical factors, plus exploring the role of ASM. Taken together, the data indicate that some of the poor perinatal outcomes previously reported in pregnant WWE are at least in part attributable to confounders and most importantly, that much of the adverse outcomes appear to be linked with prenatal ASM exposure. Yet, we were not able to account for important epilepsy characteristics that influence the choice of ASM, such as epilepsy type and syndrome and seizure frequency and severity. Prescribing ASMs during pregnancy and to people of childbearing potential should be weighed very carefully against the effects of withdrawal of ASM while planning pregnancy or indeed during pregnancy. The Mothers and Babies: Reducing Risk through Audits and Confidential Enquiries across the UK's Saving Lives, Improving Mothers’ Care 2023 report found 14 deaths due to sudden unexpected death in epilepsy that was double the 2013–2015 rate. In addition, it was unclear whether some of the WWE who died had been adherent to their ASM during pregnancy.^48^ Outside of pregnancy, there is an increased morbidity associated with valproate withdrawal in young adults with epilepsy, even if they switch to another ASM.^49^ The restrictions on prenatal ASM medication is not only for valproate.^50^, ^51^ The increased odds of induced labor compared to pregnant women without epilepsy could reflect careful and cautious management of pregnancies, including the perinatal period, in WWE in our nationwide study population. A recent American prospective multicenter study found that WWE had similar cesarean section rates compared to pregnant controls, but the study recruitment hospitals were tertiary academic centers and, thus, findings may not be representative of the general population. Of note, the authors found that in non‐recruitment hospitals, which were mainly community‐based institutions, cesarean rates without labor were more than two‐fold higher among WWE than among controls. The authors concluded that differences in perinatal expertise might have contributed to the observed differences in cesarean section rates. Our findings of increased odds of induced labor in WWE would be consistent with UK national guidelines that WWE should receive pre‐pregnancy counseling at epilepsy diagnosis and regularly during management, including preconception counseling on the risk of ASM use during pregnancy to offspring.^52^ However, the current study highlights that women without epilepsy who are taking prenatal ASM are also at increased odds for adverse perinatal outcomes. It will be of much interest to see how our results align with an ongoing study (https://publichealthscotland.scot/publications/medicines‐in‐pregnancy‐scotland‐wide‐cohort‐study‐protocol/). We propose that advice about ASM use during pregnancy should come from a relevant specialist managing the woman's underlying condition alongside an obstetrician. The specialist may be an epileptologist in the case of WWE but could be a psychiatrist or neurologist in WWoE.

The increase in congenital conditions in offspring with valproate and carbamazepine exposure is similar to that reported in other studies, but our finding of a lack of association with topiramate is surprising and counter to previous published evidence.^13^, ^53^ This may be because the number of pregnancies with topiramate exposure in the current study was low, and we did not have data on spontaneous abortions that may be related to congenital conditions. Regarding maternal and neonatal/infant death, these data are limited by a low frequency of events and could not be modeled. Furthermore, we did not have details of the cause or circumstances surrounding the deaths, and any increased odds for death could be related to the underlying condition for which the specific ASM was prescribed. However, we had no information on the indication; lamotrigine and valproate can be prescribed for psychiatric reasons.^10^ It is also feasible that they were taking the ASM for seizure control but that it was either ineffective during pregnancy or there was a problem with adherence.^54^ Further research on individual ASMs in pregnancy and perinatal outcomes is urgently needed, and so are investigations into outcomes for infants, (particularly in light of recent evidence linking prenatal exposure to specific ASMs), and at different doses, with offspring neurodevelopmental and neuropsychiatric conditions.^55^, ^56^

### 4.1 Strengths and limitations

The main strengths of this study are the large, national sample, and over a decade of available data on women with and without epilepsy. We were unable to account for epilepsy characteristics such as etiology, type/syndrome, or seizure frequency/severity.^57^ The definition of maternal epilepsy in the current study aimed to identify women where epilepsy was a concern for their pregnancy. Nonetheless it is possible that some individuals in the group of WWE without prenatal ASM exposure were misclassified as WWE in general. Some individuals could have had resolved epilepsy, such as those with childhood absence epilepsy that remitted, or they might have never had epilepsy, for example, those with functional seizures, convulsive syncope, provoked seizures, or other coding error. In addition, we are only able to identify women with epilepsy if this has been recorded on their pregnancy recorded (SMR02) or if they have been admitted to hospital with epilepsy (SMR01). Therefore, those women whose epilepsy was not recorded on either SMR01 or SMR02 could have been incorrectly assigned to the women without epilepsy group. Yet, the accuracy of SMR02 and SMR01 is 90%, and identifying epilepsy using routinely collected health data in Scotland has been validated.^31^

We were unable to account for whether the individual had received a short course (acute) or long‐term daily maintenance ASM treatment, and we did not have information concerning the trimester of dispensation. It could be argued that primidone and tiagabine, although included in the ASM list and previously used in other research,^33^ are not often used in epilepsy management and should not have been included in our modeling. However, the number of pregnant women taking these ASMs was small (<10 for both) and thus, in effect, did not contribute substantially to the modeling. Furthermore, data were limited in the analyses for some ASM monotherapies, and we were unable to undertake comparisons between ASM types, doses, combinations, or polytherapy, as indicated earlier in the study methods.

We inadvertently omitted folate^58^ and maternal chronic comorbidities (that could be used to determine a Charlson comorbidity index) such as psychiatric conditions/intellectual disability^59^, ^60^, ^61^, ^62^ as covariates. Similarly, we did not investigate other reasons for ASM use aside from epilepsy. These are major limitations that could not be addressed in the time scale of the project but the lessons learned have helped to inform an ongoing study on ASM in pregnancy in which these additional variables are being investigated (https://publichealthscotland.scot/publications/medicines‐in‐pregnancy‐scotland‐wide‐cohort‐study‐protocol/). Finally, we also dichotomized age as younger than 35 or 35 years of age or over, which is reasonable considering the known risks associated with advanced maternal age in pregnancy^3^, ^4^; yet a more granular categorization by age might have had different results, particularly given that adolescent pregnancies also carry some increased obstetric risks such as small for gestational age^63^ and neonatal intraventricular hemorrhage.^4^ Limitations of the current study raise the possibility of unmeasured confounding. Some results were suppressed due to disclosure prevention measures, and some outcomes were not modeled due to low event frequencies, necessitating further research with a larger sample.

### 4.2 Implications for future research

The pressing need for research on individual ASMs in pregnancy and perinatal outcomes has been highlighted earlier. The relationship between maternal epilepsy, prenatal ASM exposure, and perinatal outcomes is multifaceted and likely influenced by ASM type/dose,^13^, ^17^, ^47^ characteristics such as epilepsy type/syndrome,^64^, ^65^, ^66^ and comorbidities such as psychiatric/mental health conditions^60^, ^61^, ^62^ more prevalent in WWE.^59^ These factors, along with the reason for prescribing ASMs to WWoE, were not investigated, and attempts should be made to include this in future work. In addition, studying the longer‐term outcomes beyond the perinatal period, such as offspring neurodevelopmental, educational, and socioeconomic outcomes, would provide valuable insight into the overall impact of maternal epilepsy and prenatal ASM exposure. Research into models of care and prescribers and the effect on outcomes is also needed.

## 5 CONCLUSIONS

Maternal epilepsy is associated with many adverse perinatal outcomes, but many of these may be linked primarily with prenatal ASM exposure. Because women can take ASM for reasons other than epilepsy, physicians other than epileptologists, such as psychiatrists who prescribe ASM for reasons other than for seizure control, need to be aware of this association in order to counsel women appropriately. We postulate that joint care between obstetricians and experts who may prescribe ASM could improve perinatal outcomes.

## AUTHOR CONTRIBUTIONS

Mr. Paolo Pierino Mazzone: conceptualization, project administration, formal analysis, investigation, methodology, validation, visualization, writing–original draft, and writing–review and editing. Dr. Mairead Black: validation, writing–review and editing, and supervision. Professor Richard F. M. Chin: conceptualization, formal analysis, investigation, methodology, validation, resources, visualization, writing–review and editing, funding acquisition, and supervision. Professor Christopher J. Weir: conceptualization, formal analysis, investigation, methodology, validation, resources, visualization, writing–review and editing, and supervision. Dr. Jacqueline Stephen: conceptualization, formal analysis, investigation, methodology, validation, resources, visualization, and supervision. Dr. Sohinee Bhattacharya: conceptualization, methodology, resources, and supervision.

## FUNDING INFORMATION

This research received no specific grant from funding agencies in the public, commercial, or not‐for‐profit sectors.

## CONFLICT OF INTEREST STATEMENT

Dr. Black is a funded co‐investigator on the National Institute for Health Research (NIHR) Episafe project. Professor Chin reported consultant, lecture, and/or conference‐attendance fees from Eisai, GWPharmaceuticals, and Zogenix. Dr. Bhattacharya reported grants from the Medical Research Council outside the submitted work. Other authors have no conflicts of interest to disclose. We confirm that we have read the Journal's position on issues involved in ethical publication and affirm that this report is consistent with those guidelines. The data are not publicly available due to privacy restrictions.

## Supporting information


Appendix S1.

## Data Availability

Restrictions apply to the availability of the data that support the findings of this study, which are only available through application to the relevant authorities, namely, the Electronic Data Research and Innovation Service of Public Health Scotland (https://publichealthscotland.scot/resources‐and‐tools/health‐intelligence‐and‐data‐management/electronic‐data‐research‐and‐innovation‐service‐edris/overview/what‐is‐edris/). Applications for access to the data are then reviewed before approval by the Public Benefit and Privacy Panel for Health and Social Care (HSC‐PBPP).
