# Supplementary material for: Association of maternal epilepsy with perinatal outcomes, and an exploration of prenatal antiseizure medication: A population‐based retrospective cohort study
Source: Epilepsia. 2025 Jun 16;66(9):3353–68. doi: 10.1111/epi.18484 (PMC12455434; doi:10.1111/epi.18484)
Supplement: Supplementary file 1 — Appendix S1. [file EPI-66-3353-s001.docx]

**Supplemental Material**

**Association of maternal epilepsy with perinatal outcomes, and an exploration of prenatal antiseizure medication**

**Contents**

[Figure S1. Data linkage process 2](#_Toc196378255)

[Table S1. Variables and datasets 5](#_Toc196378256)

[Table S2. List of anti-seizure medications used in analysis 13](#_Toc196378257)

[Table S3. Association between covariates and exposures: Odds Ratio (OR) and 95% Confidence Interval. 14](#_Toc196378258)

[Table S4. Sensitivity analysis: Odds of neonatal congenital conditions excluding chromosomal anomalies comparing women with and without epilepsy. 15](#_Toc196378259)

[Table S5. Sensitivity analysis: Odds of perinatal outcomes comparing women with and without epilepsy using extended prenatal anti-seizure medication exposure period. 16](#_Toc196378260)

[Table S6. Sensitivity analysis: Odds of perinatal outcomes comparing complete case, best-worst case, and multiple imputation results in women with vs without epilepsy. 18](#_Toc196378261)

# Figure S1. Data linkage process

**
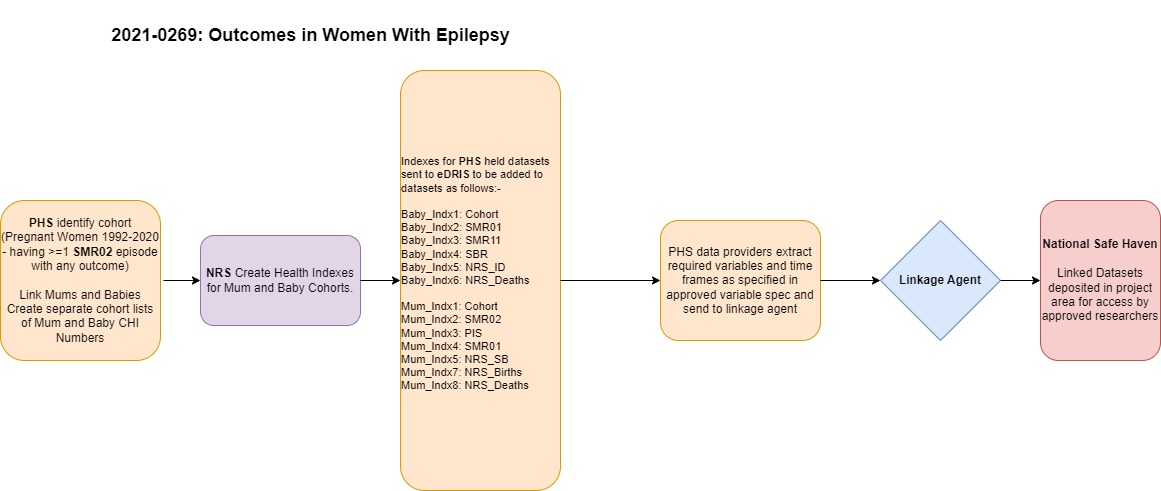
**

This figure depicts the flow of data from data controllers to the National Safe Haven where approved researchers securely access anonymised data. Data are held securely by data controllers, and a request is put forward by a team of researchers for access to data. A cohort is then defined based on the data access request which will detail the study population and all study variables, including which datasets they are stored in. This then enables the data controller to use the unique patient identifier (community health index number – CHI) for each individual and link their records between different health datasets. Furthermore, any child born will have their own CHI number which is linked to their mother from birth. This allows for mother-child linkage across datasets. Data providers then extract the required variables based on the data access application from the approved researchers, and this is then sent to the linkage agent (eDRIS) who links all the data and delivers to the National Safe Haven where the approved researchers can access and conduct their research.

**Abbreviations:** SMR01 – Acute Inpatient/day case dataset; SMR02 – Maternity Inpatient/day case dataset; SMR11 – The neonatal inpatient record; SBR – Scottish Birth Record; NRS – National Records of Scotland; PIS – Prescribing Information System; PHS - Public Health Scotland.

# Table S1. Variables and datasets

| **Exposure** | **Derived functional form of variable and comments** | **Dataset** | **ICD-10 codes (1997-2021)** |
| --- | --- | --- | --- |
| **Maternal epilepsy** | Yes or No  Identified using ICD codes on the SMR02 and hospital admission with an epilepsy diagnosis on the SMR01. | SMR02  SMR01 | G40 and G41 including available subcategories: "G400", "G400 D", "G401", "G402", "G403", "G404", "G405", "G406", "G407", "G408", "G409", "G410", "G411", "G412", "G418", "G419" |
| **Any prenatal ASM** | Yes or No  “The prenatal period was defined as the interval between pregnancy end date (livebirth, stillbirth, maternal death during pregnancy) and 30 days prior to the recorded estimated gestation on the pregnancy record database (SMR02) variable minus 2 weeks (estimated conception date)”.  The ASM was identified using the medication name variable, pre-identified by eDRIS using British National Formulary codes (<https://openprescribing.net/bnf/0408/>). Please see **Table S2** for details of ASMs.  Any prenatal ASM was defined as any monotherapy or polytherapy dispensed within the prenatal period. | PIS | Not applicable |
| **Covariates** | **Derived functional form of variable and comments** | **Dataset** | **ICD-10 codes (1997-2021)** |
| **Maternal age** | <35 years or >=35  Identified using the variable specifying the maternal age in years during pregnancy. | SMR02 | Not applicable |
| **Previous pregnancies** | Yes or No  “Yes” refers to one or more previous pregnancies identified from the Parity variable (calculated as total previous pregnancies, not including previous spontaneous or therapeutic abortions). | SMR02 | Not applicable |
| **Previous caesarean section** | Yes or No  “Yes” refers to one or more previous caesarean sections based on the variable identifying the total number of previous caesarean sections. | SMR02 | Not applicable |
| **Foetal/neonatal sex** | Male or Female  Identified using the sex of the baby variable. | SMR02 | Not applicable |
| **Deprivation decile** | Ordinally categorised as 1-10 based on the variable specifying the Scottish Index of Multiple Deprivation decile of the individual, wherein 1 is the most deprived and 10 is the least deprived. | SMR02 | Not applicable |
| **Pre-existing hypertensive disorders** | Yes or No  Identified using ICD-10 codes | SMR02 | O10 including available subcategories:  "O100", "O101", "O102", "O103", "O104", "O109" |
| **Pre-existing diabetes** | Yes or No  Identified using pre-existing diabetes variable and ICD-10 codes on the pregnancy record. | SMR02 | O24 including available subcategories:  "O240", "O240 D", "O241", "O241 D", "O242", "O243", "O243 D" |
| **Body mass index** | Normal (18.5-24)  Obese (30-60)  Overweight (25-29)  Underweight (17.5-18.4)  Calculated based on weight of mother (kg) and height of mother (m)  (W/H^2^). Weight of mother is recorded at first booking. Data outside of these ranges coded as missing (<17.5 BMI & >60 BMI) | SMR02 | Not applicable |
| Illicit drugs or their substitute or Drug Misuse during pregnancy | Yes or No  Based on the variable identifying self-reported drug misuse at any time during the current pregnancy. This includes illegal drugs, solvents and gases, drugs prescribed for someone else, and drugs prescribed by a doctor as a substitute for addiction or to alleviate withdrawal symptoms. | SMR02 | Not applicable |
| **Smoking during pregnancy** | Yes or No  Based on the variable identifying smoking during pregnancy by the individual. | SMR02 | Not applicable |
| **Maternal and foetal outcomes** | **Original Format** | **Dataset** | ICD-10 codes (1997-2021) |
| **Induction of labour** | Yes or No  Based on induction of labour variable. | SMR02 | Not applicable |
| **Preterm Birth** | Yes or No  Based on estimated gestation <37 weeks from the estimated gestational age variable and ICD-10 codes | SMR02 | O60.1 and O60.3 including available subcategories:  "O601", "O603", "O60X” |
| **Caesarean Section** | Yes or No  Identified using the mode of delivery variable and ICD 10 codes | SMR02 | O82 including available subcategories:  "O820", "O821", "O822", "O828", "O829" |
| **Stillbirth** | Yes or No  Identified using the outcome of pregnancy variable in the SMR02 dataset. | SMR02 | Not applicable |
| **Gestational Hypertension** | Yes or No  Identified using ICD codes. | SMR02 | O13 including available subcategories:  "O13”, O13X", "O139" |
| **Eclampsia** | Yes or No  Identified using ICD codes. | SMR02 | O15 including available subcategories:  "O15", "O150", "O151", "O152", "O159" |
| **Preeclampsia** | Yes or No  Identified using ICD codes. | SMR02 | O14 including available subcategories:  "O14", "O140", "O141", "O142", "O149" |
| **Gestational Diabetes** | Yes or No  Identified using the diabetes variable and ICD codes in the SMR02 dataset. | SMR02 | O24.4 including available subcategories:  "O244" |
| **Antepartum haemorrhage** | Yes or No  Identified using ICD codes. | SMR02 | O20 and O46 including available subcategories:  "O20", "O200", "O208", "O209", "O460", "O468", "O469" |
| **Postpartum haemorrhage** | Yes or No  Identified using ICD codes. | SMR02 | O72 including available subcategories:  "O72", "O720", "O721", "O722", "O723" |
| **Placental abruption** | Yes or No  Identified using ICD codes. | SMR02 | 045 including available subcategories:  "O45", "O450", "O458", "O459" |
| **Placenta Praevia** | Yes or No  Identified using ICD codes. | SMR02 | O440-O445  including available subcategories:  "O44", "O440", "O441" |
| **Maternal death during pregnancy and up to 1-year postpartum** | Yes or No  Identified using deaths variable in the NRS Deaths dataset.  Death during pregnancy or plus one year after delivery. | NRS Deaths | Not applicable |
| **Neonatal outcomes** | **Original Format** | **Dataset** | ICD-10 codes (1997-2021) |
| **Congenital conditions** | Yes or No  Identified using ICD codes in the SMR11, SBR, SM01 and SMR02 datasets. | SMR11  SBR  SMR01  SMR02 | **Q00-Q99 including available subcategories:**  "Q00", "Q000", "Q001", "Q002", "Q01", "Q010", "Q011", "Q012", "Q018", "Q019", "Q02", "Q02X", "Q03", "Q030", "Q031", "Q038", "Q039", "Q04", "Q040", "Q041", "Q042", "Q043", "Q044", "Q045", "Q046", "Q048", "Q049", "Q05", "Q050", "Q051", "Q052", "Q053", "Q054", "Q055", "Q056", "Q057", "Q058", "Q059", "Q06", "Q060", "Q061", "Q062", "Q063", "Q064", "Q068", "Q069", "Q07", "Q070", "Q078", "Q079", "Q10", "Q100", "Q101", "Q102", "Q103", "Q104", "Q105", "Q106","Q107", "Q11", "Q110", "Q111", "Q112", "Q113", "Q12", "Q120", “Q121", "Q122", "Q123", "Q124", "Q128", "Q129", "Q13", "Q130", “Q131", "Q132", "Q133", "Q134", "Q135", "Q138", "Q139", "Q14", “Q140", "Q141", "Q142", "Q143", "Q148", "Q149", "Q15", "Q150", "Q158", "Q159", "Q16", "Q160", "Q161", "Q162", "Q163", "Q164", "Q165", "Q169", "Q17", "Q170", "Q171", "Q172", "Q173", "Q174",”Q175", "Q178", "Q179", "Q18", "Q180", "Q181", "Q182", "Q183", ”Q184", "Q185", "Q186", "Q187", "Q188", "Q189", "Q20", "Q200", "Q201", "Q202", "Q203", "Q204", "Q205", "Q206", "Q208", "Q209", "Q21", "Q210", "Q211", "Q212", "Q213", "Q214", "Q218", "Q219", “Q22", "Q220", "Q221", "Q222", "Q223", "Q224", "Q225", "Q226", "Q228", "Q229", "Q23", "Q230", "Q231", "Q232", "Q233", "Q234", "Q238", "Q239", "Q24", "Q240", "Q241", "Q242", "Q243", "Q2442", "Q245", "Q246", "Q248", "Q249", "Q25", "Q250", "Q251", "Q252", "Q253", "Q254", "Q255", "Q256", "Q257", "Q258", "Q259", "Q26", "Q260", "Q261", "Q262", "Q263", "Q264", "Q265", "Q266", "Q268", "Q269", "Q27", "Q270", "Q271", "Q272", "Q273", "Q274", "Q278", "Q279", "Q28", "Q280", "Q281", "Q282", "Q283", "Q288", "Q289", “Q30", "Q300", "Q301", "Q302", "Q303", "Q308", "Q309", "Q31", "Q310", "Q311", "Q312", "Q313", "Q314", "Q315", "Q318", "Q319", ”Q32", "Q320", "Q321", "Q322", "Q323", "Q324", "Q33", "Q330", "Q331", "Q332", "Q333", "Q334", "Q335", "Q336", "Q338", "Q339", “Q34", "Q340", "Q341", "Q348", "Q349", "Q35", "Q350", "Q351", "Q352", "Q353", "Q354", "Q355", "Q356", "Q357", "Q358", "Q359", "Q36", "Q360", "Q361", "Q369", "Q37", "Q370", "Q371", "Q372", "Q373", "Q374", "Q375", "Q378", "Q379", "Q38", "Q380", "Q381", "Q382", "Q383", "Q384", "Q385", "Q386", "Q387", "Q388", "Q39", “Q390", "Q391", "Q392", "Q393", "Q394", "Q395", "Q396", "Q398", "Q399", "Q40", "Q400", "Q401", "Q402", "Q403", "Q408", "Q409", "Q41", "Q410", "Q411", "Q412", "Q418", "Q419", "Q42", "Q420", “Q421", "Q422", "Q423", "Q428", "Q429", "Q43", "Q430", "Q431", “Q432", "Q433", "Q434", "Q435", "Q436", "Q437", "Q438", "Q439", “Q44", "Q440", "Q441", "Q442", "Q443", "Q444", "Q445", "Q446", "Q447", "Q45", "Q450", "Q451", "Q452", "Q453", "Q458", "Q459", ”Q50", "Q500", "Q501", "Q502", "Q503", "Q504", "Q505", "Q506", "Q51", "Q510", "Q511", "Q512", "Q513", "Q514", "Q515", "Q516", "Q517", "Q518", "Q519", "Q52", "Q520", "Q521", "Q522", "Q523", "Q524", "Q525", "Q526", "Q527", "Q528", "Q529", "Q53", "Q530", "Q531", "Q532", "Q539", "Q54", "Q540", "Q541", "Q542", "Q543", "Q544", "Q548", "Q549", "Q55", "Q550", "Q551", "Q552", "Q553", "Q554", "Q555", "Q556", "Q558", "Q559", "Q56", "Q560", "Q561", "Q562", "Q563", "Q564", "Q60", "Q600", "Q601", "Q602", "Q603", "Q604", "Q605", "Q606", "Q61", "Q610", "Q611", "Q612", "Q613", "Q614", "Q615", "Q618", "Q619", "Q62", "Q620", "Q621", "Q622", "Q623", "Q624", "Q625", "Q626", "Q627", "Q628", "Q63", "Q630", "Q631", "Q632", "Q633", "Q638", "Q639", "Q64", "Q640", "Q641", "Q642", "Q643", "Q644", "Q645", "Q646", "Q647", "Q648", "Q649", "Q65", "Q650", "Q651", "Q652", "Q653", "Q654", "Q655", "Q656", "Q658", "Q659", "Q66", "Q660", "Q661", "Q662", "Q663", "Q664", "Q665", "Q666", "Q667", "Q668", "Q669", "Q67", "Q670", "Q671", "Q672", "Q673", "Q674", "Q675", "Q676", "Q677", "Q678", "Q68", "Q680", "Q681", "Q682", "Q683", "Q684", "Q685", "Q688", "Q69", "Q690", "Q691", "Q692", "Q699", "Q70", "Q700", "Q701", "Q702", "Q703", "Q704", "Q709", "Q71", "Q710", "Q711", "Q712", "Q713", "Q714", "Q715", "Q716", "Q718", "Q719", "Q72", "Q720", "Q721", "Q722", "Q723", "Q724", "Q725", "Q726", "Q727", "Q728", "Q729", "Q73", "Q730", "Q731", "Q738", "Q74", "Q740", "Q741", "Q742", "Q743", "Q748", "Q749", "Q75", "Q750", "Q751", "Q752", "Q753", "Q754", "Q755", "Q758", "Q759", "Q76", "Q760", "Q761", "Q762", "Q763", "Q764", "Q765", "Q766", "Q767", "Q768", "Q769", "Q77", "Q770", "Q771", "Q772", "Q773", "Q774", "Q775", "Q776", "Q777", “Q778", "Q779", "Q78", "Q780", "Q781", "Q782", "Q783", "Q784", “Q785", "Q786", "Q788", "Q789", "Q79", "Q790", "Q791", "Q792", "Q793", "Q794", "Q795", "Q796", "Q798", "Q799", "Q80", "Q800", "Q801", "Q802", "Q803", "Q804", "Q808", "Q809", "Q81", "Q810", "Q811", "Q812", "Q818", "Q819", "Q82", "Q820", "Q821", "Q822", "Q823", "Q824", "Q825", "Q828", "Q829", "Q83", "Q830", "Q831", "Q832", "Q833", "Q838", "Q839", "Q84", "Q840", "Q841", "Q842", "Q843", "Q844", "Q845", "Q846", "Q848", "Q849", "Q85", "Q850", "Q851", "Q858", "Q859", "Q86", "Q860", "Q861", "Q862", "Q868", "Q87", "Q870", "Q871", "Q872", "Q873", "Q874", "Q875", "Q878", "Q89", "Q890", "Q891", "Q892", "Q893", "Q894", "Q897", "Q898", "Q899", "Q90", "Q900", "Q901", "Q902", "Q909", "Q91", "Q910", "Q911", "Q912", "Q913", "Q914", "Q915", "Q916", "Q917", "Q92", "Q920", "Q921", "Q922", "Q923", "Q924", "Q925", "Q926", "Q927", "Q928", "Q929", "Q93", "Q930", "Q931", "Q932", "Q933", "Q934", "Q935", "Q936", "Q937", "Q938", "Q939", "Q95", "Q950", "Q951", "Q952", "Q953", "Q954", "Q955", "Q958", "Q959", "Q96", "Q960", "Q961", "Q962", "Q963", "Q964", "Q968", "Q969", "Q97", "Q970", "Q971", "Q972", "Q973", "Q978", "Q979", "Q98", "Q980", "Q981", "Q982", "Q983", "Q984", "Q985", "Q986", "Q987", "Q988", "Q989", "Q99", "Q990", "Q991", "Q992", "Q998", "Q999", "Q00", "Q01", "Q02", "Q03", "Q04", "Q041", "Q042", "Q8703", "Q040", "Q040b", "Q0400", "Q05", "Q06", "Q07", "Q8703", "Q10", "Q110", "D111", "Q112", "Q11", "Q12", "Q120", "Q13", "Q14", "Q15", "Q150", "Q16", "Q160", "Q161", "Q17", "Q18", "Q20", "Q200", "Q211", "Q250", "Q201", "Q202", "Q203", "Q204", "Q205", "Q206", "Q21", "Q212", "Q213", "Q214", "Q2182", "Q22", "Q220", "Q224", "Q225", "Q226", "Q23", "Q230", "Q232", "Q234", "Q24", "Q242", "Q244", "Q245", "Q251", "Q252", "Q253", "Q262", "Q263", "Q25", "Q26", “Q300", "Q32", "Q33", "Q34", "Q3380", "Q35", "Q36", "Q37", "Q38", "Q39", "Q40", "Q41", "Q42", "Q43", "Q44", "Q45", "Q790", "Q390", "Q391", "Q410", "Q411", "Q412", "Q418", "Q420", "Q421", "Q422", "Q423", "Q431", "Q442", "Q451", "Q433", "Q792", "Q793", "Q795", "Q60", "Q61", "Q62", "Q63", "Q64", "Q794", "Q600", "Q601", "Q606", "Q614b", "Q6140", "Q6141", "Q620", "Q621", "Q623", "Q631", "Q632", "Q640", "Q641", "Q642b", "Q6420", "Q794", "Q50", "Q51", "Q52", "Q53", "Q54", "Q55", "Q56", "Q65", "Q66", "Q67", "Q68", "Q69", "Q70", "Q71", "Q72", "Q73", "Q74", "Q710", "Q712", "Q7180", "Q720", "Q722", "Q7280", "Q730", "Q7131", "Q714", "Q7231", "Q725", "Q715", "Q726", "Q716", "Q727", "Q711", "Q721", "Q731", "Q660", "Q650", "Q651", "Q652", "Q69", "Q70", "P350", "P351", "P354", "P358", "P371", "Q0435", "Q206", "Q240", "Q3381", "Q411", "Q412", "Q418", "Q710", "Q712", "Q7180", "Q720", "Q722", "Q7280", "Q730", "Q750", "Q793", "Q7980", "Q7982", "Q86", "Q8706", "Q8708", "Q8724", "Q8726", "Q890", "Q893", "Q894", "Q8980", "Q750", "Q4471", "Q6190", "Q7402", "Q7484", "Q751", "Q754", "Q7581", "Q77", "Q780", "Q781", "Q782", "Q783", "Q784", "Q785", "Q786", "Q788", "Q789", "Q796", "Q80", "Q81", "Q820", "Q821", "Q822", "Q823", "Q824", "Q8282", "Q8283", "Q850", "Q851", "Q8581", "Q87", "Q8934", "Q90", "Q91", "Q92", "Q93", "Q96", "Q97", "Q98", "Q99", "D821", "Q770", "Q771", "Q772", "Q778", "Q914", "Q915", "Q916", "Q917", "Q910", "Q911", "Q912", "Q913", "Q927"  **Restricted list excludes chromosomal anomalies (Q90-99 including available subcategories):**  "Q00", "Q000", "Q001", "Q002", "Q01", "Q010", "Q011", "Q012", "Q018","Q019", "Q02", "Q02X", "Q03", "Q030", "Q031", "Q038", "Q039", "Q04", "Q040", "Q041", "Q042", "Q043", "Q044", "Q045", "Q046", "Q048", "Q049", "Q05", "Q050", "Q051", "Q052", "Q053", "Q054", "Q055", "Q056", "Q057", "Q058", "Q059", "Q06", "Q060", "Q061", "Q062", "Q063", "Q064", "Q068", "Q069", "Q07", "Q070", "Q078", "Q079", "Q20", "Q200", "Q201", "Q202", "Q203", "Q204", "Q205", "Q206", "Q208", "Q209", "Q21", "Q210", "Q211", "Q212", "Q213", "Q214", "Q218", "Q219", "Q22", "Q220", "Q221", "Q222", "Q223", "Q224", "Q225", "Q226", "Q228", "Q229", "Q23", "Q230", "Q231", "Q232", "Q233", "Q234", "Q238", "Q239", "Q24", "Q240", "Q241", "Q242", "Q243", "Q2442", "Q245", "Q246", "Q248", "Q249", "Q25", "Q250", "Q251", "Q252", "Q253", "Q254", "Q255", "Q256", "Q257", "Q258", "Q259", "Q26", "Q260", "Q261", "Q262", "Q263", "Q264", "Q265", "Q266", "Q268", "Q269", "Q35", "Q350", "Q351", "Q352", "Q353", "Q354", "Q355", "Q356", "Q357", "Q358", "Q359", "Q36", "Q360", "Q361", "Q369", "Q37", "Q370", "Q371", "Q372", "Q373", "Q374", "Q375", "Q378", "Q379", "Q65", "Q650", "Q651", "Q652", "Q653", "Q654", "Q655", "Q656", "Q658", "Q659", "Q66", "Q660", "Q661", "Q662", "Q663", "Q664", "Q665", "Q666", "Q667", "Q668", "Q669", "Q67", "Q670", "Q671", "Q672", "Q673", "Q674", "Q675", "Q676", "Q677", "Q678", "Q68", "Q680", "Q681", "Q682", "Q683", "Q684", "Q685", "Q688", "Q69", "Q690", "Q691", "Q692", "Q699", "Q70", "Q700", "Q701", "Q702", "Q703", "Q704", "Q709", "Q71", "Q710", "Q711", "Q712", "Q713", "Q714", "Q715", "Q716", "Q718", "Q719", "Q72", "Q720", "Q721", "Q722", "Q723", "Q724", "Q725", "Q726", "Q727", "Q728", "Q729", "Q73", "Q730", "Q731", "Q738", "Q74", "Q740", "Q741", "Q742", "Q743", "Q748", "Q749", "Q866", "Q8703", "Q8680", "P350", "P351", "P354", "P358", "P371” |
| **Neonatal intensive care unit admission** | Yes or No  Identified using admission to neonatal intensive care unit variable in the SMR02 dataset. | SMR02 | Not applicable |
| **Low birth weight (<2500g)** | Yes or No  Identified based on the weight of the offspring variable measured in grams, wherein LBW <2500 grams. | SMR02 | Not applicable |
| **Neonatal/Infant Death** | Yes or No  Identified using the death variable in the NRS Infant deaths dataset. | NRS Infant Deaths | Not applicable |
| **Apgar 5-mins <7** | Yes or No  Based on Apgar score variable (1-10) | SMR02 | Not applicable |

**Abbreviations:** SMR01 – Acute Inpatient/day case dataset; SMR02 – Maternity Inpatient/day case dataset; ICD – International Classification of Disease; SMR11 – The neonatal inpatient record; SBR – Scottish Birth Record; NRS – National Records of Scotland; PIS – Prescribing Information System; ASM: Antiseizure medication.

# Table S2. List of anti-seizure medications used in analysis

| **Prenatal ASMs** | **The five most frequently dispensed medications** |
| --- | --- |
| Brivaracetam | -- |
| Carbamazepine | Carbamazepine |
| Clobazam | -- |
| Eslicarbazepine | -- |
| Ethosuximide | -- |
| Lacosamide | -- |
| Lamotrigine | Lamotrigine |
| Levetiracetam | Levetiracetam |
| Oxcarbazepine | -- |
| Perampanel | -- |
| Phenobarbital | -- |
| Phenytoin | -- |
| Primidone | -- |
| Sodium valproate | Sodium valproate |
| Tiagabine | -- |
| Topiramate | Topiramate |
| Vigabatrin | -- |
| Zonisamide | -- |

# Table S3. Association between covariates and exposures: Odds Ratio (OR) and 95% Confidence Interval.

| Characteristic | WWoE | WWE |
| --- | --- | --- |
| Epilepsy Diagnosis  Yes | Reference | Not applicable |
| Maternal age  >=35 years | Reference | 0.81 [0.72, 0.91] |
| Previous pregnancies  Yes >=1 | Reference | 1.08 [0.99, 1.18] |
| Previous caesarean section  Yes | Reference | 1.12 [0.99, 1.27] |
| Foetal/neonatal sex  Female | Reference | 0.95 [0.87, 1.04] |
| Deprivation*  10 | Reference | 0.57 [0.46, 0.70] |
| Pre-existing hypertensive disorders  Yes | Reference | 2.09 [1.20, 3.34] |
| Pre-existing diabetes  Yes | Reference | 1.44 [0.92, 2.14] |
| BMI  Obese | Reference | 1.20 [1.08, 1.33] |
| Illicit drugs or their substitute or Drug Misuse during pregnancy  Yes | Reference | 3.10 [2.48, 3.81] |
| Smoker during pregnancy  Yes | Reference | 1.84 [1.66, 2.03] |

**Abbreviations**: WWE: Women with epilepsy WWoE: Women without epilepsy; with ASMs: All pregnancies exposed to any prenatal ASM; without ASMs: All pregnancies unexposed to any prenatal ASM; TPR – Topiramate; CBZ – Carbamazepine; LVT – Levetiracetam; LMT – Lamotrigine; SVP – Sodium Valproate; BMI: Body mass index.

* Deprivation for individual ASM monotherapies based on the Scottish Index of Multiple Deprivation 2020 - Scotland level population-weighted decile (1=most deprived; 10=least deprived).

# Table S4. Sensitivity analysis: Odds of neonatal congenital conditions excluding chromosomal anomalies comparing women with and without epilepsy.

| Outcome | WWoE (N=627,178) | WWE (N=2,022) | OR [95CI] | aOR [95CI]_a_ | aOR [95CI]_b_ | aOR [95CI]_c_ |
| --- | --- | --- | --- | --- | --- | --- |
| Congenital conditions* | 4459 | 22 | 1.53 [1.00, 2.34] | 1.43 [0.94, 2.19] | 0.93 [0.56, 1.54] | 1.06 [0.58, 1.93] |

**Abbreviations**: WWoE: Women without epilepsy; WWE: Women with epilepsy; OR: Odds Ratio; aOR: Adjusted Odds Ratio; 95CI: 95% Confidence Interval. aOR_a_ Adjusted for maternal age, parity, previous caesarean sections, foetal/neonatal sex, deprivation, pre-existing hypertensive disorders, pre-existing diabetes (Initial adjustment); aOR_b_ Initial adjustment plus any prenatal ASM; aOR_c_ Initial adjustment plus any prenatal ASM, BMI, Illicit drugs or their substitute or Drug Misuse during pregnancy , Smoker during pregnancy.

*Congenital conditions include any congenital condition as identified using ICD-10 codes, excluding those with chromosomal anomalies (please see Table).

# Table S5. Sensitivity analysis: Odds of perinatal outcomes comparing women with and without epilepsy using extended prenatal anti-seizure medication exposure period.

|  | Prenatal ASM | | | | Prenatal +6 months pre-pregnancy | | | |
| --- | --- | --- | --- | --- | --- | --- | --- | --- |
| Maternal and Foetal Outcomes | **OR [95CI]** | **aOR [95CI]_a_** | **aOR [95CI]_b_** | **aOR [95CI]_c_** | **OR [95CI]** | **aOR [95CI]_a_** | **aOR [95CI]_b_** | **aOR [95CI]_c_** |
| Preterm Birth | **1.68 [1.44, 1.97]** | **1.56 [1.33, 1.83]** | 1.07 [0.88, 1.29] | 1.09 [0.87, 1.37] | **1.71 [1.47, 1.98]** | **1.58 [1.36, 1.84]** | 1.13 [0.95, 1.35] | 1.12 [0.91, 1.38] |
| Induced labour | **1.51 [1.37, 1.66]** | **1.51 [1.37, 1.67]** | **1.17 [1.04, 1.31]** | **1.17 [1.02, 1.34]** | **1.49 [1.36, 1.63]** | **1.49 [1.35, 1.64]** | **1.16** **[1.04, 1.30]** | **1.20** **[1.05, 1.36]** |
| Caesarean Section | **1.28 [1.09, 1.51]** | **1.15 [1.03, 1.29]** | 1.02 [0.90, 1.17] | 1.05 [0.90, 1.23] | **1.31 [1.13, 1.53]** | **1.16 [1.04, 1.30]** | 0.99 [0.88, 1.12] | 1.02 [0.89, 1.18] |
| Stillbirth | n.c. *(n<10)* | n.c. *(n<10)* | n.c. *(n<10)* | n.c. *(n<10)* | n.c. *(n<10)* | n.c. *(n<10)* | n.c. *(n<10)* | n.c. *(n<10)* |
| Gestational Hypertension | **0.68 [0.46, 1.00]** | 0.68 [0.45, 1.01] | 0.79 [0.50, 1.24] | 0.83 [0.51, 1.37] | 0.70 [0.48, 1.01] | 0.70 [0.48, 1.02] | 0.70 [0.46, 1.06] | 0.76 [0.48, 1.20] |
| Eclampsia | n.c. *(n<10)* | n.c. *(n<10)* | n.c. *(n<10)* | n.c. *(n<10)* | n.c. *(n<10)* | n.c. *(n<10)* | n.c. *(n<10)* | n.c. *(n<10)* |
| Preeclampsia | **1.42 [1.00, 2.02]** | **1.43 [1.01, 2.03]** | **1.57 [1.02, 2.41]** | 1.36 [0.80, 2.31] | **1.46 [1.05, 2.04]** | **1.48 [1.06, 2.06]** | **1.59 [1.07, 2.35]** | 1.37 [0.85, 2.20] |
| Gestational Diabetes | **1.30 [1.02, 1.66]** | 1.22 [0.95, 1.56] | 1.13 [0.84, 1.51] | 0.95 [0.68, 1.34] | **1.31 [1.04, 1.64]** | 1.21 [0.96, 1.53] | 1.10 [0.84, 1.43] | 0.98 [0.71, 1.34] |
| APH | 1.10 [0.72, 1.68] | 1.08 [0.71, 1.65] | 0.95 [0.58, 1.56] | 0.95 [0.53, 1.72] | 1.05 [0.69, 1.58] | 1.03 [0.68, 1.56] | 1.04 [0.65, 1.66] | 1.03 [0.59, 1.79] |
| PPH | 1.10 [0.99, 1.22] | 1.08 [0.97, 1.19] | 1.08 [0.93, 1.18] | 1.07 [0.93, 1.24] | **1.13 [1.02, 1.24]** | 1.10 [1.00, 1.21] | 1.04 [0.93, 1.16] | 1.07 [0.94, 1.22] |
| Placental abruption | n.c. *(n<10)* | n.c. *(n<10)* | n.c. *(n<10)* | n.c. *(n<10)* | n.c. *(n<10)* | n.c. *(n<10)* | n.c. *(n<10)* | n.c. *(n<10)* |
| Placenta Praevia | n.c. *(n<10)* | n.c. *(n<10)* | n.c. *(n<10)* | n.c. *(n<10)* | n.c. *(n<10)* | n.c. *(n<10)* | n.c. *(n<10)* | n.c. *(n<10)* |
| Maternal Deaths | n.c. *(n<10)* | n.c. *(n<10)* | n.c. *(n<10)* | n.c. *(n<10)* | n.c. *(n<10)* | n.c. *(n<10)* | n.c. *(n<10)* | n.c. *(n<10)* |
| Neonatal Outcomes | **OR [95CI]** | **aOR [95CI]_a_** | **aOR [95CI]_b_** | **aOR [95CI]_c_** | **OR [95CI]** | **aOR [95CI]_a_** | **aOR [95CI]_b_** | **aOR [95CI]_c_** |
| Congenital conditions | 1.07 [0.81, 1.41] | 1.01 [0.76, 1.34] | 0.76 [0.55, 1.05] | 0.98 [0.68, 1.41] | **1.51 [1.01, 2.27]** | 1.41 [0.94, 2.12] | 0.86 [0.54, 1.37] | 1.00 [0.57, 1.75] |
| NICU | **1.43 [1.23, 1.66]** | **1.34 [1.16, 1.56]** | 0.89 [0.75, 1.07] | 0.97 [0.79, 1.20] | **1.57 [1.37, 1.81]** | **1.49 [1.30, 1.71]** | 0.98 [0.84, 1.15] | 1.03 [0.85, 1.24] |
| LBW | **1.90 [1.62, 2.23]** | **1.72 [1.46, 2.03]** | **1.22 [1.00, 1.49]** | 1.12 [0.88, 1.42] | **1.96 [1.68, 2.28]** | **1.77 [1.52, 2.07]** | **1.27 [1.06, 1.52]** | 1.12 [0.90, 1.39] |
| Neonatal/Infant Death | n.c. *(n<10)* | n.c. *(n<10)* | n.c. *(n<10)* | n.c. *(n<10)* | n.c. *(n<10)* | n.c. *(n<10)* | n.c. *(n<10)* | n.c. *(n<10)* |
| Apgar 5-mins <7 | **1.51 [1.20, 1.90]** | **1.44 [1.15, 1.81]** | 1.15 [0.87, 1.52] | 1.32 [0.96, 1.81] | **1.54 [1.24, 1.90]** | **1.47 [1.18, 1.82]** | 1.13 [0.88, 1.45] | 1.27 [0.95, 1.69] |

**Abbreviations**: WWoE: Women without epilepsy; WWE: Women with epilepsy; OR: Odds Ratio; aOR: Adjusted Odds Ratio; 95CI: 95% Confidence Interval; APH: Antepartum haemorrhage; PPH: Postpartum haemorrhage; NICU: Neonatal intensive care unit admission; LBW: Low birthweight; n.c.: not calculated to prevent overfitting due to low event frequencies. aOR_a_ Adjusted for maternal age, parity, previous caesarean sections, foetal/neonatal sex, deprivation, pre-existing hypertensive disorder, pre-existing diabetes (Initial adjustment); aOR_b_ Initial adjustment plus any prenatal ASM; aOR_c_ Initial adjustment plus any prenatal ASM, BMI, Illicit drugs or their substitute or Drug Misuse during pregnancy , Smoker during pregnancy.

# Table S6. Sensitivity analysis: Odds of perinatal outcomes comparing complete case, best-worst case, and multiple imputation results in women with vs without epilepsy.

|  | Complete-case | Best-case | Worst-case | MI data |
| --- | --- | --- | --- | --- |
| Maternal and Foetal Outcomes | **aOR_c_ [95CI]** | **aOR_c_ [95CI]** | **aOR_c_ [95CI]** | **aOR_c_ [95CI]** |
| Preterm Birth | 1.19 [0.91, 1.56] | 1.18 [0.94, 1.49] | 1.21 [0.96, 1.52] | 1.19 (0.95, 1.49) |
| Induced labour | 1.12 [0.95, 1.32] | 1.14 [0.99, 1.31] | 1.15 [1.00, 1.32] | 1.12 (0.97, 1.29) |
| Caesarean Section | 1.03 [0.87, 1.23] | 1.02 [0.88, 1.19] | 1.04 [0.89, 1.21] | 1.03 (0.89, 1.20) |
| Stillbirth | n.c. *(n<10)* | n.c. *(n<10)* | n.c. *(n<10)* | n.c. *(n<10)* |
| Gestational Hypertension | 0.86 [0.50, 1.50] | 0.97 [0.59, 1.59] | 0.95 [0.58, 1.56] | 0.98 (0.60, 1.69) |
| Eclampsia | n.c. *(n<10)* | n.c. *(n<10)* | n.c. *(n<10)* | n.c. *(n<10)* |
| Preeclampsia | 1.38 [0.76, 2.49] | 1.85 [1.14, 3.00] | 1.80 [1.11, 2.93] | 1.87 (1.15, 3.03) |
| Gestational Diabetes | 0.85 [0.55, 1.33] | 1.20 [0.83, 1.73] | 1.16 [0.80, 1.67] | 1.21 (0.84, 1.75) |
| APH | 1.29 [0.65, 2.57] | 1.12 [0.61, 2.04] | 1.10 [0.60, 2.01] | 1.11 (0.61, 2.03) |
| PPH | 1.06 [0.90, 1.25] | 1.07 [0.93, 1.23] | 1.07 [0.93, 1.23] | 1.07 (0.93, 1.23) |
| Placental abruption | 3.03 [0.99, 9.28] | 2.47 [0.85, 7.15] | 2.50 [0.86, 7.24] | 2.49 (0.86, 7.21) |
| Placenta Praevia | n.c. *(n<10)* | n.c. *(n<10)* | n.c. *(n<10)* | n.c. *(n<10)* |
| Maternal Deaths | n.c. *(n<10)* | n.c. *(n<10)* | n.c. *(n<10)* | n.c. *(n<10)* |
| Neonatal Outcomes | **aOR_c_ [95CI]** | **aOR_c_ [95CI]** | **aOR_c_ [95CI]** | **aOR_c_ [95CI]** |
| Congenital conditions | 1.15 [0.76, 1.73] | 0.93 [0.64, 1.35] | 0.94 [0.65, 1.37] | 0.93 (0.64, 1.35) |
| NICU | 0.97 [0.76, 1.26] | 0.86 [0.69, 1.08] | 0.89 [0.71, 1.11] | 0.87 (0.70, 1.09) |
| LBW | 1.27 [0.96, 1.67] | 1.35 [1.08, 1.70] | 1.39 [1.10, 1.74] | 1.36 (1.08, 1.71) |
| Neonatal/Infant Death | n.c. *(n<10)* | n.c. *(n<10)* | n.c. *(n<10)* | n.c. *(n<10)* |
| Apgar 5-mins <7 | 1.30 [0.88, 1.91] | 1.15 [0.81, 1.63] | 1.15 [0.81, 1.63] | 1.16 (0.82, 1.64) |

**Abbreviations**: WWoE: Women without epilepsy; WWE: Women with epilepsy; aOR: Adjusted Odds Ratio; 95CI: 95% Confidence Interval; APH: Antepartum haemorrhage; PPH: Postpartum haemorrhage; NICU: Neonatal intensive care unit admission; LBW: Low birthweight; n.c.: not calculated to prevent overfitting due to low event frequencies. aOR_c_ Adjusted for maternal age, parity, previous caesarean sections, foetal/neonatal sex, deprivation, pre-existing hypertensive disorders, pre-exiting diabetes, BMI, Illicit drugs or their substitute or Drug Misuse during pregnancy , smoker during pregnancy, any prenatal ASM exposure.
